# Astrocytic FABP7 Alleviates Depression‐Like Behaviors of Chronic Unpredictable Mild Stress Mice by Regulating Neuroinflammation and Hippocampal Spinogenesis

**DOI:** 10.1096/fj.202403417RR

**Published:** 2025-05-07

**Authors:** Zihui Geng, Fanzhen Peng, Ziqian Cheng, Jingyun Su, Jinfang Song, Xu Han, Runxin Li, Xin Li, Ranji Cui, Bingjin Li

**Affiliations:** ^1^ Jilin Provinicial Key Laoratory on Molecular and Chemical Genetic Sencond Hospital of Jilin University Changchun People's Republic of China; ^2^ Engineering Lab on Screening of Antidepressant Drugs Jilin Province Development and Reform Commission Changchun People's Republic of China

**Keywords:** antidepressant, Cav‐1, FABP7, neuroinflammation, spinogenesis

## Abstract

Fatty acid binding protein 7 (FABP7) is prominently expressed in astrocytes and is a critical regulator of inflammatory responses. Accumulating evidence suggests that FABP7 is crucial in neuropsychological disease through the modulation of spinogenesis. Nonetheless, the impact of FABP7 on depressive disorders and the underlying mechanisms is not fully understood. Here, we investigated the antidepressant properties of FABP7 using the chronic unpredictable mild stress (CUMS)‐induced model of depression and possible mechanisms. Our results revealed that depressive‐like behavior induced by CUMS was associated with decreased levels of FABP7 protein in the hippocampus (HP). Furthermore, the overexpression of FABP7 in the HP mitigated the depressive‐like behavior and increased the expression of its downstream target caveolin‐1 (Cav‐1). FABP7 overexpression in the HP specifically regulates the expression of the astrocyte marker protein GFAP, as well as the blood–brain barrier (BBB)‐associated proteins AQP4, CLDN‐5, occludin, and LRP1. Notably, the CUMS‐induced upregulation of the pro‐inflammatory factors IL‐1β and IL‐6 was also significantly reversed by FABP7 overexpression in the HP. This intervention also led to increased levels of postsynaptic proteins, including PSD95 and GluA1, as well as an increase in brain‐derived neurotrophic factor (BDNF) and enhanced neuronal dendritic spine density. The findings indicate that FABP7 exerts antidepressant‐like properties by inhibiting inflammation, regulating spinogenesis, and modulating BBB‐related proteins.

## Graphical abstract

Schematic diagram of the FABP7 prevents depression‐like behaviors by inhibiting inflammation, regulating spinogenesis, and regulating BBB‐related proteins.

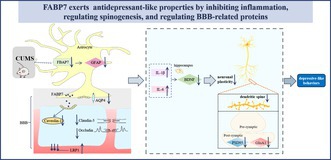

## 1 Introduction

As one of the most prevalent psychological diseases worldwide, depression is characterized primarily by symptoms such as persistently depressed mood, reduced social interest, and cognitive slowing, which can lead to suicide in severe cases [1]. Current estimates indicate that approximately 350 million individuals are affected by depression worldwide, and morbidity is rising annually [2, 3]. Therefore, it is of great significance to research the pathophysiological mechanism of depression in depth, in order to identify new targets for more efficacious depression treatments.

Astrocytes, the most plentiful and pervasive glial cells in the brain, play an essential function in sustaining brain homeostasis. Extensive research has demonstrated that astrocytes are involved in depressive behaviors through diverse mechanisms, including participation in the nervous system's inflammatory responses [4, 5], modulation of synaptic plasticity [6], and the construction and preservation of the blood–brain barrier (BBB) [7]. The astrocyte‐abundant brain‐type fatty acid binding protein, FABP7, serves as an intracellular transporter of fatty acid. This small molecular protein, with a molecular weight of 14 kDa, is highly expressed in astrocytes and functions to regulate inflammatory responses. FABP7‐knockout (FABP7‐KO) in mice led to an increase in the mRNA levels of IL‐17 and TNF‐α within the lesioned regions in a mouse model of multiple sclerosis (MS) [8]. Additionally, FABP7 regulates neurogenesis [9, 10], spinogenesis [11], and astrocyte proliferation [12, 13]. In the medial prefrontal cortex (mPFC), the lack of FABP7 in astrocytes leads to aberrant dendritic morphology and reduces the density of dendritic spines on pyramidal neurons [11]. Collectively, this literature shows that FABP7 is intimately connected with the pathological and physiological mechanisms underlying neurodegenerative diseases and neuropsychiatric disorders [14, 15, 16, 17].

As a downstream target of the FABP7, studies have demonstrated that the protein expression of caveolin‐1 (Cav‐l, a major structural protein of caveolae) is significantly decreased in astrocytes of FABP7‐KO mice [18]. This suggests that FABP7 may regulate lipid raft function by modulating downstream Cav‐1 expression, thereby mediating the response of astrocytes to lipopolysaccharide (LPS) stimulation [18, 19]. Caveolae are pits on the surface of the cell membrane, where Cav‐1 is an essential scaffolding protein. Cav‐1 regulates multiple signaling pathways, including those related to oxidative stress [20], inflammation‐induced tissue damage [21], cellular apoptosis [22, 23], and lipid metabolism [24]. As a fundamental constituent of caveolae, Cav‐1 facilitates the transport of peptides to the brain and modulates the permeability of the BBB [25]. In a model of traumatic brain injury (TBI), FABP7 regulates BBB function in part by modulating Cav‐1 expression. This modulation affects the intracerebral environment and the stability of the BBB [26], suggesting that Cav‐1 may serve as a target gene of FABP7. However, the influence of FABP7 in depression triggered by stress, along with the related mechanisms, remains unclear.

In this study, behavioral tests were utilized to evaluate behavior. Western blotting, immunofluorescence staining, co‐immunoprecipitation, and Golgi‐Cox staining were employed to investigate the effect and mechanism of FABP7 in depression.

## 2 Materials and Methods

### 2.1 Animals

Adult male ICR mice (6–8 weeks old, 23–27 g) were provided by Jilin University and housed in a standard environment (12 h light/dark cycle, temperature kept at 24°C ± 1°C, humidity maintained at 40%–50%), with each cage accommodating a single mouse. A three‐day environmental acclimatization period preceded the experiments, during which mice had unrestricted availability to food and water. All procedures adhered to the guidelines specified in the Laboratory Animal Guidance for Ethical Review of Animal Welfare (GB/T 35892‐2018).

### 2.2 CUMS

Consistent with previous literature [27, 28], this study constructed a depression model in mice exposed to CUMS for 2 weeks. CUMS mice were randomly assigned to one long‐duration stressor and one short‐duration stressor daily for 2 weeks. The long‐duration stressors were: (a) water deprivation for 24 h, (b) food deprivation for 24 h, (c) continuous light exposure for 36 h, (d) exposure to damp bedding for 24 h, and (e) cage tilting at 45° for 16 h. The short‐duration stressors were: (a) forced swimming at 4°C for 5 min, (b) footshock for 15 min, (c) restraint for 2 h, (d) cage shaking for 5 min (with variable directions to the extent that the mice could not maintain their balance), and (e) olfactory stimulation for 4 h (placing the mice in a cage sprinkled with pepper but devoid of bedding). The same stimulus was not utilized on consecutive days.

### 2.3 Surgery

Mice received an intraperitoneal injection of pentobarbital sodium at a dosage of 65 mg/kg for anesthesia. The coordinates of the hippocampal brain area were determined in reference to the brain atlas [29]. The control virus (ov‐control: gfaABC1D promoter‐MCS‐EGFP‐WPRE‐bGH PolyA, 1.78E+13 v.g/mL, Genechem, Shanghai, China) or FABP7 construct (ov‐FABP7: gfaABC1D promoter‐FABP7‐EGFP‐WPRE‐bGH PolyA, 2.13E+13 v.g/mL, Genechem, Shanghai, China) was delivered bilaterally through microinjection in the hippocampus (HP, −1.8 mm anterior–posterior, ±1.5 mm medial‐lateral, and‐2.0 mm dorsal‐ventral from bregma; Figure 3A) at a rate of 0.5 μL/min, with a unilateral volume of 1 μL.

### 2.4 Behavioral Tests

#### 2.4.1 Open Field Test (OFT)

The apparatus consists of an opaque, dark plastic open‐topped cylinder featuring a diameter of 48.8 cm and a height of 16 cm. The bottom surface of the cylinder is divided into 16 equal‐sized areas by three concentric circles. Each circle is further divided into essentially equal areas. The circles have diameters of 12 cm (divided into 1 region), 29.4 cm (divided into 5 regions), and 48 cm (divided into 10 zones). Mice to be tested were positioned in the central area of the arena's bottom surface and permitted to move freely for 6 min. Both horizontal movements (the number of times all four paws crossed into the new grid) and vertical movements (the number of times a mouse stood on its two hind legs) were recorded using a video camera. Behavioral outcomes were assessed by an evaluator unaware of the experimental treatments.

#### 2.4.2 Forced Swimming Test (FST)

The experimental apparatus comprised a transparent, cylindrical container (11 cm diameter and 35 cm high) filled with water (24°C–26°C) to a depth of 25 cm. Mice were carefully placed into the water, and their movements were captured by a video recorder for 6 min. The initial 2 min were considered the acclimatization period, and the subsequent 4 min were used to document the behavior of the mice. Immobility was defined as floating with only those movements of the limbs imperative to prevent the head from submerging, in addition to the duration of active behaviors (swimming, climbing, and struggling). Behavioral outcomes were assessed by an evaluator unaware of the experimental treatments.

#### 2.4.3 Tail Suspension Test (TST)

During the test, the rear 1/3 of each mouse's tail was wrapped with tape, with the excess tape affixing the mouse to an elevated stand, with the mouse's head elevated 45 cm above the table surface below. A video camera was used to record the activity of the mice for 6 min. The first 2 min of the experiment was the acclimatization period, and the immobilization time (stationary limbs or with only minimal limb movements) was recorded and calculated for the last 4 min. Behavioral outcomes were assessed by an evaluator unaware of the experimental treatments.

#### 2.4.4 Sucrose Preference Test (SPT)

Two days of acclimatization training for the mice with two bottles of 2% sucrose solution were required before the formal test [30]. Mice were subjected to a 12‐h deprivation of both water and food, after which the SPT was conducted. Each mouse was supplied with one bottle containing a 2% sucrose solution and another bottle of purified water, both with known initial weights. After 3 h, all bottles were taken out and weighed. The sucrose preference (the proportion of sucrose solution consumption to the overall liquid consumption) was calculated. Behavioral outcomes were assessed by an evaluator unaware of the experimental treatments.

### 2.5 Western Blotting

Total protein was extracted with RIPA lysate containing 1% PMSF from hippocampal tissue (*n* = 6). Following the execution of 10%–15% SDS‐PAGE, targeted proteins were transferred to polyvinylidene fluoride (PVDF) membranes, which were incubated with the listed primary antibodies at 4°C overnight: FABP7 (1:1000, rabbit monoclonal; CST, Danvers, MA, USA, #13347); Cav‐1 (1:1000, rabbit monoclonal; CST, #3267); AQP4 (1:1000, mouse monoclonal; Santa Cruz Biotechnology, CA, USA, #sc‐32739); CLDN‐5 (1:1000; mouse monoclonal; Thermo Fisher Scientific, Waltham, MA, USA, #35‐2500); occludin (1:1000, mouse monoclonal; Thermo Fisher Scientific, #33‐1500); LRP1 (1:2000, rabbit monoclonal; Abcam, Cambridge, UK, #ab92544); GFAP (1:1000, mouse monoclonal; CST, #3670); BDNF (1:1000, rabbit polyclonal; ABclonal, Wuhan, China, #A16229); PSD95 (1:1000, mouse monoclonal; Abcam, #ab192757); GluA1 (1:2000, rabbit polyclonal; Abcam, #ab109450); Synapsin (1:1000, rabbit polyclonal; Abcam, #ab64581); NeuN (1:1000, mouse monoclonal; Abcam, #ab104224); IL‐1β (1:1000, rabbit polyclonal; Abcam, #ab2105); IL‐6 (1:1000, rabbit polyclonal; Proteintech, Wuhan, China, #21865‐1‐AP); or β‐actin (1:2000, mouse monoclonal; Transgen Biotech, Beijing, China, #HC201). After washing with TBST (TBS containing 0.1% Tween‐20), the membrane was incubated with secondary antibodies (Anti‐rabbit: 1:3000, ZSBG‐Bio, Beijing, China, #ZB2301; or anti‐mouse: 1:3000, ZSBG‐Bio, #ZB2305) for 1 h at room temperature. After washing with TBST, the developer (WBKLS0500, Millipore) was uniformly dripped onto the membrane, and the analysis was conducted using a film developer. Subsequently, the grayscale values were quantified with Image J software, and the data analysis was conducted utilizing GraphPad Prism software.

### 2.6 Immunofluorescent Staining

After the behavioral experiment, whole brains were extracted (*n* = 3). Serial frozen sections with a thickness of 15 μm were obtained along the coronal plane and were air‐dried at room temperature. Following a wash with PBS (0.01 mol/L), the sections were transferred to a blocking buffer for 2 h at normal temperature. Next, alternate sections were incubated overnight at 4°C with the following primary antibodies: FABP7 (1:100, rabbit monoclonal; CST, #13347), GFAP (1:1000, mouse monoclonal; CST, #3670), and NeuN (1:1000, mouse monoclonal; Abcam, #ab104224) (antibodies were prepared with PBS containing 0.15% TritonX‐100 and 1.5% goat serum). After washing with PBS, the sections were treated for 1 h at normal temperature with the following fluorophore‐conjugated secondary antibodies: Alexa Fluor488 Goat Anti‐Mouse (1:200, ab150113, Abcam) or Cy3 Goat Anti‐Rabbit (1:200, AS007, ABclonal Technology, Wuhan, China), as appropriate. After DAPI (1 μg/mL (1×), sigma) staining for 5 min, an antifade Mounting Medium (Beyotime, #P0126) was used for mounting. The results were visualized with a microscope (IX73/BX51WI, Olympus).

### 2.7 Golgi‐Cox Staining

The procedures for Golgi‐Cox staining follow a previous report published by this research group [31]. After the behavioral experiment, whole brains (*n* = 3) were removed after cardiac perfusion with normal saline and immersed in Golgi‐Cox reagent for 2 days at room temperature, which consisted of 200 mL 5% potassium dichromate solution (Sinopharm Chemical Reagent Co. Ltd., Shanghai, China), 200 mL 5% mercuric chloride solution (Tongren Chemical Plant, Tongren, Guizhou, China), 160 mL 5% potassium chromate solution (Tianjin BASF Chemical Trade Co. Ltd., Tianjin, China), and 400 mL double distilled water (ddH_2_O). The treatment was replaced with a new Golgi‐Cox reagent solution and continued for 14 days at normal temperature. After that, the brain tissues were transferred in a sucrose solution gradient (10%, 20%, and 30%) at 4°C. Sections of 200 μm thickness were obtained on adherent slides using a vibrating slicer and dried at room temperature away from light. The staining steps included alkalinization with ammonia for 60 min, fixation with fixative (consisted of 1.25% potassium dichromate solution, 0.115% mercuric chloride solution) for 30 min, gradient dehydration with ethanol (50%, 70%, 95%, 100%, and again 100%, for 1 min at each concentration), and xylene for 4 min (repeated three times). Finally, the slices were covered with neutral gum (HUSHI, 10004160) and dried at room temperature. Dendrites with a clear structure and less intersection with other dendrites were photographed using a microscope 100× oil lens. Dendritic spine density is expressed as the dendritic spine number per 10‐μm dendrite length. We counted eight neurons from each subregion of HP (CA1, CA3, and DG) in each mouse, quantitatively analyzed the dendritic branches emanating from the secondary dendrite in the neuronal apical tree, and calculated the mean of the number of dendritic spine density in each group.

### 2.8 Co‐Immunoprecipitation

The steps for CO‐IP were conducted in accordance with the instructions provided in the immunoprecipitation kit (Absin, Shanghai, China, #abs955). The steps for preparing tissue lysate were consistent with the Western blotting experiments described above. 1 μg antibody (Cav‐1, rabbit monoclonal, CST, #3267; or Rabbit IgG, rabbit polyclonal, Absin, #abs20035) was introduced to the supernatant, and the solution was gently mixed overnight at 4°C to allow for adequate antigen–antibody binding. 5 μL of Protein A and 5 μL of Protein G agarose microbeads were introduced and gently mixed overnight at 4°C, and the precipitate was retained. The precipitate was thoroughly washed with 0.5 mL of 1× wash buffer and repeated three times. The washed agarose beads were resuspended thoroughly in 40 μL 1× SDS loading buffer, heated to 95°C for 5 min, and centrifuged to obtain supernatant. The supernatant was applied to an SDS‐polyacrylamide gel and analyzed using Western blotting methods.

### 2.9 Experimental Design

To analyze the changes in FABP7 expression and elucidate the underlying mechanisms of depressive‐like behaviors, we divided the mice into a control (CON) group and a CUMS group randomly. Following the CUMS procedure, behavioral experiments (OFT, FST, TST, SPT, *n* = 9 for each group) were conducted to validate the production of depressive behavioral phenotypes. Brain tissue samples (HP, *n* = 6) and whole brains (*n* = 3) were collected for subsequent analysis.

To evaluate how FABP7 overexpression in the HP affects depressive‐like behaviors of CUMS mice, the virus (control or FABP7‐overespression) was injected into the HP and sustainably expressed for 3 weeks. Mice were then randomly assigned to a control group or a CUMS group. Following CUMS, depression‐like behaviors were evaluated through behavioral experiments (OFT: *n* = 8–15; FST: *n* = 14–15; TST: *n* = 12–14; SPT: *n* = 12–13); brain tissue samples (HP, *n* = 6) and whole brains (*n* = 6) were collected for subsequent analysis.

### 2.10 Statistical Analysis

GraphPad Prism software, version 8.0 (GraphPad Software, USA) was employed for data analysis. Data were expressed as mean ± standard deviation (SD) and normalized to the control group (CON/CON+AAV‐GFP) before analysis. The Shapiro–Wilk test was used to evaluate the normality of the data by SPSS (version 26). Comparisons between two means (CON vs. CUMS) were analyzed by two‐tailed unpaired *t*‐tests, and differences between treatment groups of more than two were analyzed by the two‐way ANOVA followed by Tukey's *post hoc* tests (factor 1: CUMS; factor 2: FABP7). *p* < 0.05 was considered statistically significant.

## 3 Results

### 3.1 CUMS Induces Depression‐Like Behavior

**FIGURE 1:**
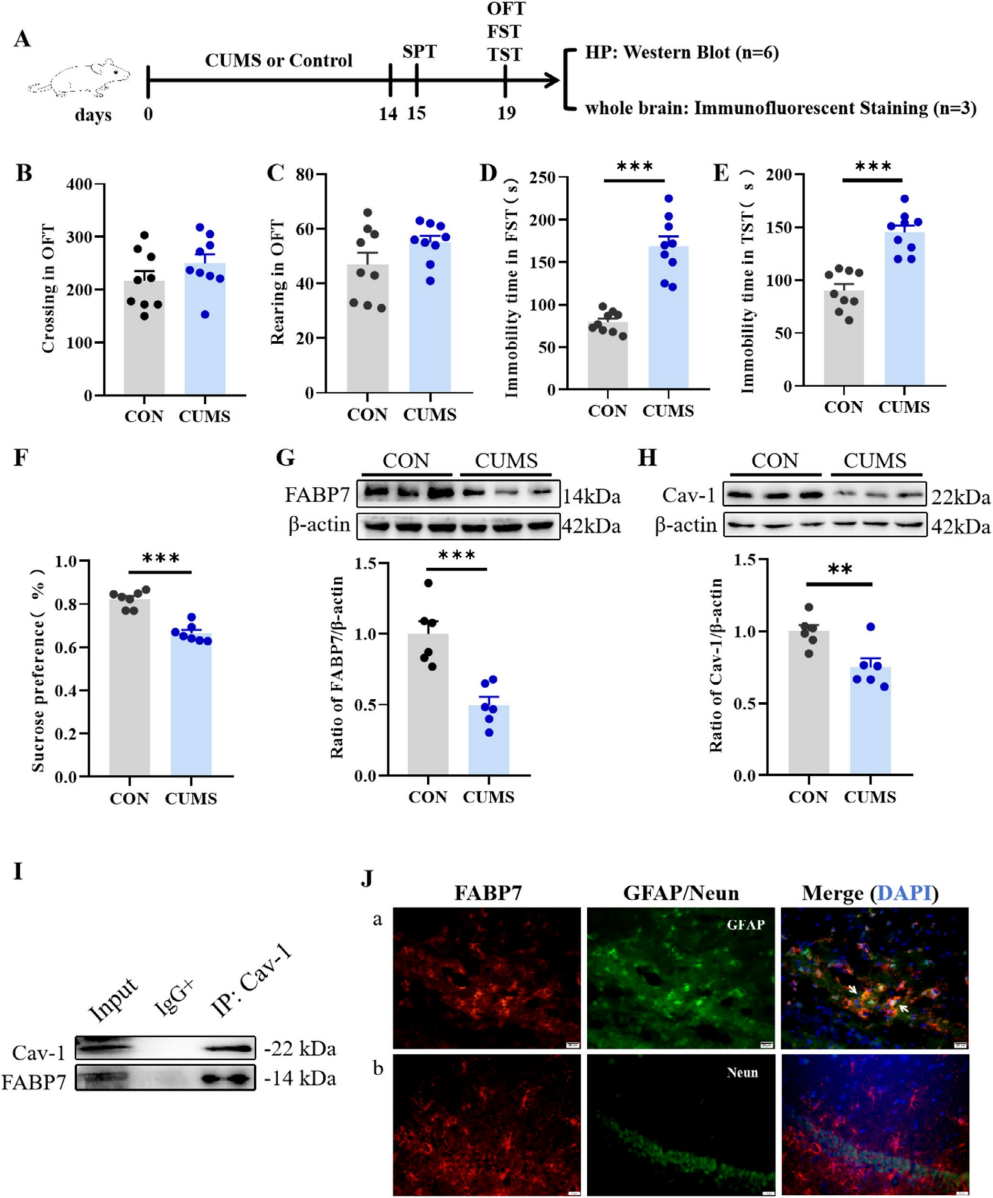
Effects of CUMS on depression‐like behavior and the expression of FABP7 and Cav‐1. (A) Schematic of the experimental procedure and schedule. (B) Number of horizontal motions in the OFT. (C) Number of vertical motions in the OFT. (D) Immobility time in the FST. (E) Immobility time in the TST. (F) Sucrose preference in the SPT. The effect of CUMS on protein expression of FABP7 (G) and Cav‐1 (H) in the HP. (I) A representative image of co‐immunoprecipitation. (J) Representative immunofluorescence double‐labeling of FABP7 and GFAP (a) /NeuN (b). Data were presented as mean ± SEM (*n* = 6–9) and normalized to the control group (CON) for analysis. Student's t test, *p < 0.05, **p < 0.01, ***p < 0.001.

To investigate whether 14‐day CUMS induces depression‐like behavior, behavioral changes were analyzed (Figure 1A). The results of the OFT showed no statistical differences in horizontal (*t*_(16)_=1.326, *p* = 0.2035; Figure 1B) or vertical (*t*_(16)_=1.630, *p* = 0.1226; Figure 1C) movements among mice across treatment groups, indicating that CUMS did not affect the exploratory behavior of the mice. The CUMS group demonstrated a statistically notable increase in immobility duration across the FST (*t*_(16)_=7.286, *p* < 0.001; Figure 1D) and TST (*t*_(16)_=6.196, *p* < 0.001; Figure 1E) relative to the control group. In the SPT, the CUMS group demonstrated a significantly diminished rate of sucrose preference relative to the control group (*t*_(12)_=7.484, *p* < 0.001; Figure 1F). Collectively, these findings indicate that CUMS exposure induces depressive‐like behaviors in mice.

### 3.2 CUMS Decreases Expression of FABP7 and Cav‐1 in HP

In order to investigate the molecular mechanisms underlying CUMS‐induced depressive‐like behaviors, changes in protein expression of FABP7 and Cav‐1 in HP were examined by Western blotting. CUMS significantly reduced the protein expression of FABP7 (*t*_(10)_=4.68, *p* < 0.001; Figure 1G) and Cav‐1 (*t*_(10)_=3.317, *p* < 0.01; Figure 1H) in HP. CO‐IP analysis indicated that FABP7 interacted with Cav‐1 in the HP (Figure 1I).

### 3.3 CUMS Decreases GFAP Expression in HP

**FIGURE 2:**
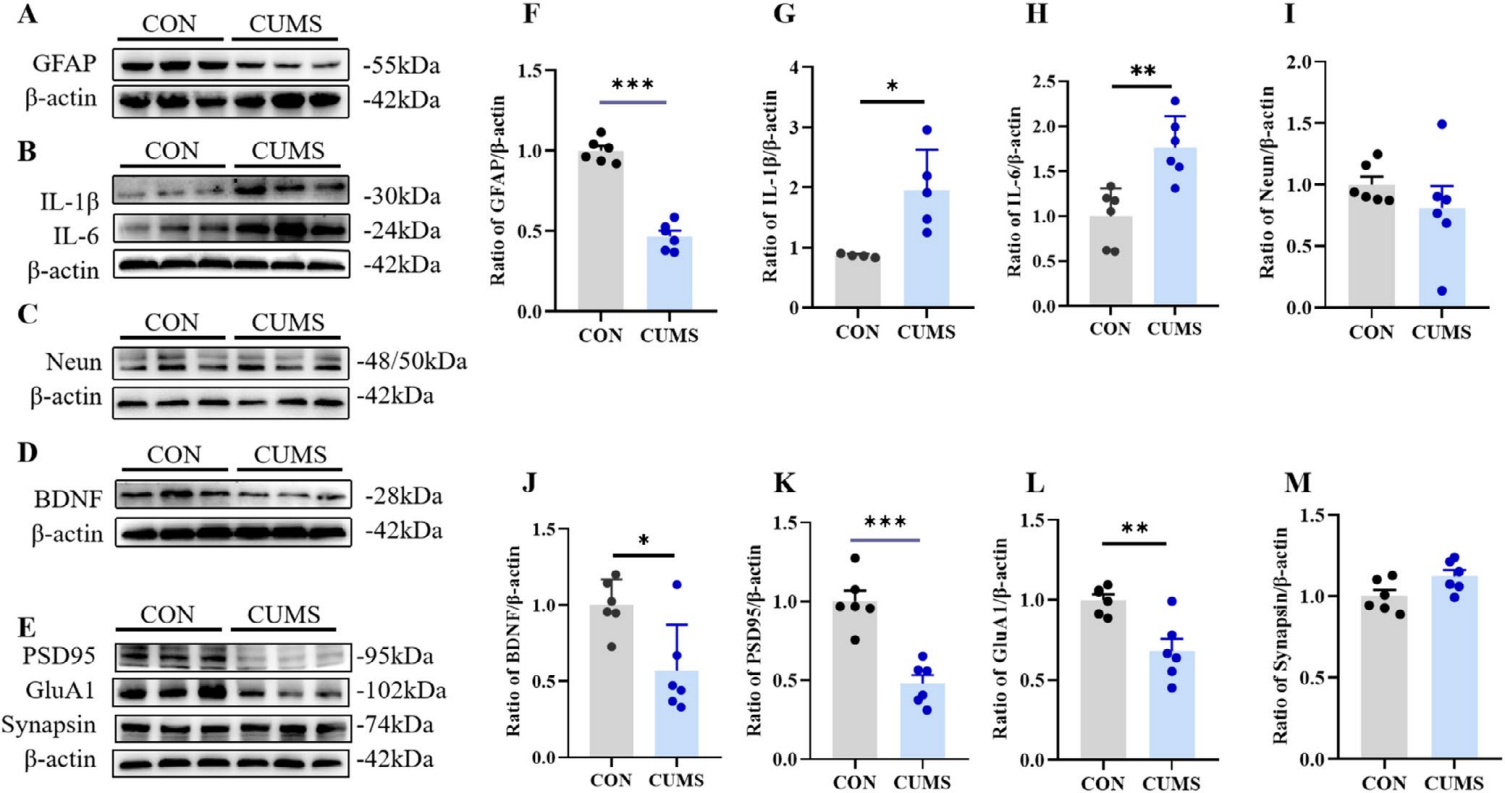
Effects of CUMS on inflammation and spinogenesis‐related proteins. (A–E) Representative western blots for GFAP (A), IL‐1β (B), IL‐6 (B), NeuN (C), BDNF (D), PSD95 (E), GluA1 (E), and synapsin (E) in the HP. (F–M) Quantitative analysis of GFAP (F), IL‐1β (G), IL‐6 (H), NeuN (I), BDNF (J), PSD95 (K), GluA1 (L), and synapsin (M) proteins in the HP. Data were presented as mean ± SEM (*n* = 5‐6) and normalized to the control group (CON) for analysis. Student's t test, *p < 0.05, **p < 0.01, ***p < 0.001.

FABP7 is mainly expressed in astrocytes in the mature brain and is not expressed in mature neuronal cells. In order to verify this phenomenon, dual immunofluorescence staining was performed to label FABP7 with GFAP, a marker protein for astrocytes, and the neuronal cytosolic protein (NeuN), a biomarker protein for mature neurons in the HP of control mice. The experimental results show that FABP7 expression overlapped with GFAP fluorescence (indicated by white arrows) but not with NeuN fluorescence (Figure 1J). These findings suggest that FABP7 is selectively and highly expressed in hippocampal astrocytes and hardly expressed in adult neuronal cells. At the same time, evidence suggests that CUMS‐triggered depressive‐like behavior is associated with changes in the expression of the astrocyte marker GFAP. Therefore, changes in GFAP expression in HP were explored by Western blotting. The results demonstrated that CUMS notably reduced the protein expression of GFAP in HP compared with the control group (*t*_(10)_=11.51, *p* < 0.001; Figure 2A,F). The above results indicate that the depressive‐like behavior produced by CUMS is related to reduced expression of GFAP in HP.

### 3.4 CUMS Regulates Levels of Inflammatory Factors in HP

Astrocytes are crucial in the activation and development of neuroinflammation, which is also associated with the occurrence of depression. Next, we assessed the expression of IL‐1β and IL‐6 through Western blotting. The results showed that CUMS elevated the protein levels of IL‐1β (*t*_(7)_=3.207, *p* < 0.05; Figure 2B,G) and IL‐6 (*t*_(10)_=4.028, *p* < 0.01; Figure 2B,H) in the HP. These findings indicate that the activation of the neuroinflammatory response is related to the emergence of depressive‐like behavior after CUMS stimulation.

### 3.5 CUMS Reduces Proteins Related to Spinogenesis in HP

Inhibition of spinogenesis is one of the mechanisms that produces depressive‐like behavior. The number of neurons, levels of neurotrophic factors, and synaptic‐related proteins are all related indicators of spinogenesis. To investigate the impact of CUMS on spinogenesis in HP, the protein levels of NeuN, brain‐derived neurotrophic factor (BDNF), and the synapse‐associated proteins PSD95, GluA1, and synapsin were measured. NeuN protein expression was not significantly changed in the HP after CUMS compared to control treatments (*t*_(10)_=0.9956, *p* = 0.3429; Figure 2C,I), while BDNF levels were significantly reduced (*t*_(10)_=3.063, *p* < 0.05; Figure 2D,J). Similarly, the expression of synapse‐associated proteins PSD95 (*t*_(10)_=5.915, *p* < 0.001; Figure 2E,K) and GluA1 (*t*_(9)_=3.795, *p* < 0.01; Figure 2E,L) was reduced in HP after CUMS, while synapsin protein expression was not significantly changed (*t*_(10)_=2.181, *p* = 0.0541; Figure 2E,M).

### 3.6 FABP7 Overexpression in HP Ameliorates CUMS‐Induced Depressive‐Like Behavior

**FIGURE 3:**
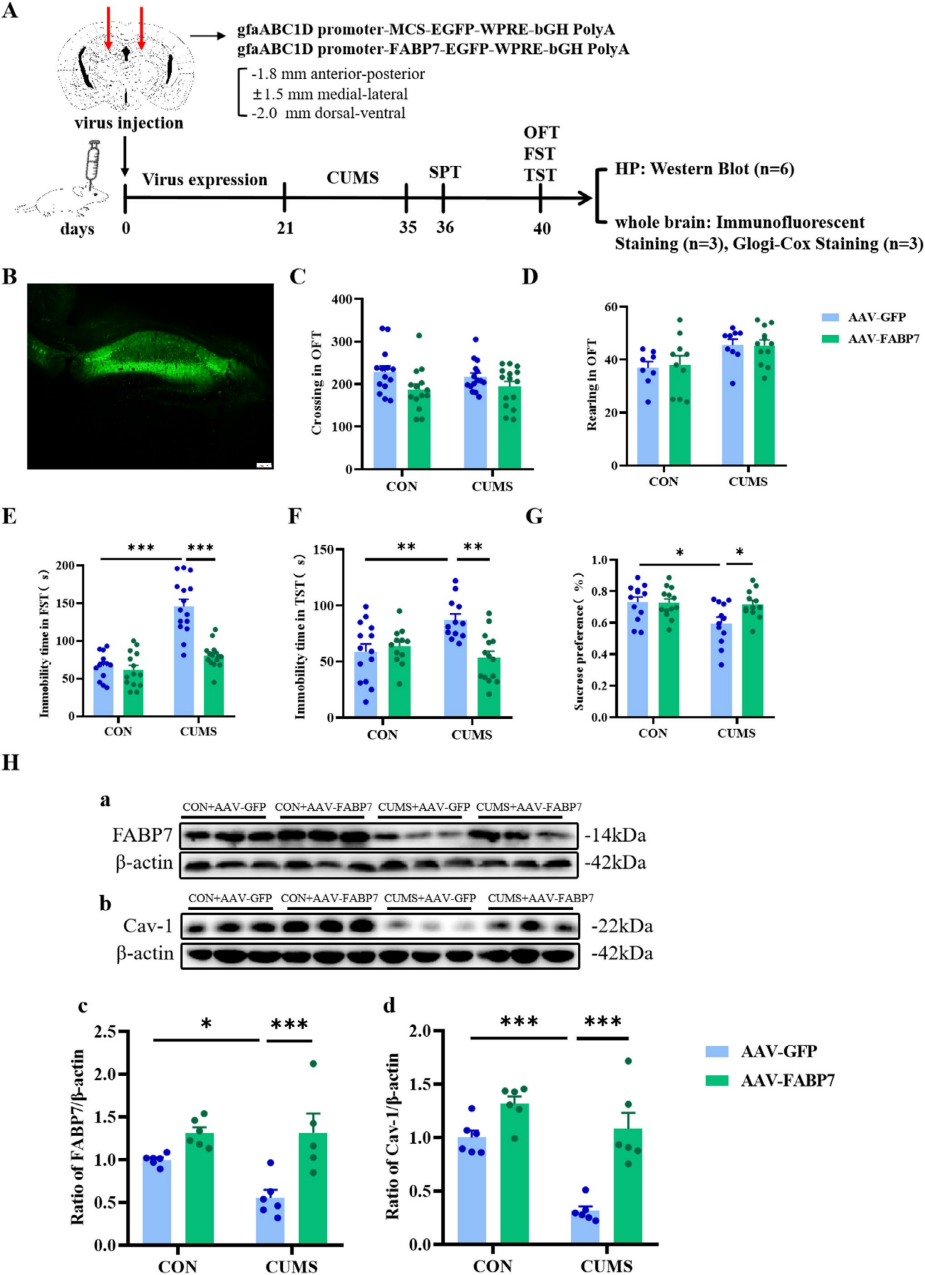
Effects of FABP7 overexpression on depression‐like behaviors and protein expressions in HP of the CUMS mice. (A) Schematic of the experimental procedure and schedule. (B) Representative fluorescent image illustrating the expression of green fluorescent protein (GFP). (C) Number of horizontal motions in the OFT. (D) Number of vertical motions in the OFT. (E) Immobility time in the FST. (F) Immobility time in the TST. (G) Sucrose preference in the SPT. (H) Changes in the protein expression of FABP7 (a, c) and Cav‐1 (b, d) in the HP of CUMS mice. Data were presented as mean ± SEM (*n* = 5–14 per group) and normalized to the control group (CON+AAV‐GFP) for analysis. Two‐way ANOVA with Tukey's honestly significant difference (HSD), *p < 0.05, **p < 0.01, ***p < 0.001.

Since CUMS caused modifications in the expression of FABP7 and associated proteins in the HP, an adeno‐associated virus producing an overexpression of FABP7 was injected bilaterally in the HP (Figure 3A,B) prior to CUMS. Behavioral tests were conducted to analyze the role of FABP7 overexpression in depressive‐like behavior resulting from CUMS (Figure 3). In the OFT, no statistically significant differences in horizontal movement were observed across the groups (*F*_CUMS(1,54)_ = 6.821, *p* < 0.05; *F*_FABP7(1,54)_ = 0.02967, *p* = 0.8693; *F*_CUMS×FABP7(1,54)_ = 0.6254, *p* = 0.4325; Figure 3C) or vertical movement (*F*_CUMS(1,35)_ = 0.04432, *p* = 0.8345; *F*_FABP7(1,35)_ = 9.615, *p* < 0.01; *F*_CUMS×FABP7(1,35)_=0.06989, *p* = 0.7930; Figure 3D) between treatment groups, indicating that FABP7 overexpression in the HP did not influence the spontaneous motor behavior after CUMS stimulation. ANOVA showed the influence of CUMS and FABP7 overexpression on immobility time in FST (*F*_CUMS(1,53)_ = 28.03, *p* < 0.001; *F*_FABP7(1,53)_ = 54.86, *p* < 0.001; *F*_CUMS×FABP7(1,53)_ = 20.76., *p* < 0.001; Figure 3E). Tukey's HSD confirmed that after FABP7 overexpression in the HP, immobility time in the FST was significantly reduced in CUMS mice (*p* < 0.001). A similar trend was seen in the immobility time of TST (*F*_CUMS(1,48)_ = 6.080, *p* < 0.05; *F*_FABP7(1,48)_ = 2.436, *p* = 0.1252; *F*_CUMS×FABP7(1,48)_ = 10.64, *p* < 0.01; Figure 3F), while the opposite results occurred for the sucrose preference (*F*_CUMS(1,45)_ = 3.362, *p* = 0.0734; *F*_FABP7(1,45)_ = 5.200, *p* < 0.05; *F*_CUMS×FABP7(1,45)_ = 3.989, *p* = 0.0519; Figure 3G) in the ANOVA. Tukey's HSD indicated that the immobility time of TST was reduced (*p* < 0.01) and the sucrose preference in SPT was increased (*p* < 0.05) in CUMS‐exposed mice after FABP7 overexpression in HP. These results suggest that FABP7 overexpression in HP can ameliorate depressive‐like behaviors induced by CUMS, including behavioral despair and anhedonia.

### 3.7 FABP7 Overexpression in HP Affects the Expression of Cav‐1 and GFAP in HP After CUMS

**FIGURE 4:**
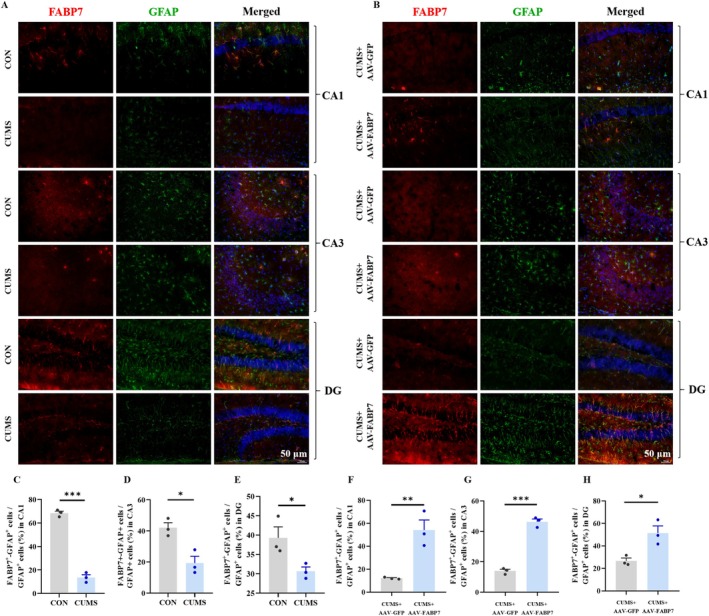
Effects of FABP7 overexpression on co‐localization of FABP7 and GFAP in CA1, CA3 and DG of the HP of the CUMS‐treated mice. (A, B) Representative images of FABP7 and GFAP of CA1, CA3, and DG regions of HP. Scale bar = 50 μm. (C–H) Quantitation of FABP7^+^‐GFAP^+^ cell in the HP. Data were presented as mean ± SEM (*n* = 3 per group) and normalized to the control group (CON/CON+AAV‐GFP) for analysis. Student's *t* test, *p < 0.05, **p < 0.01, ***p < 0.001.

**FIGURE 5:**
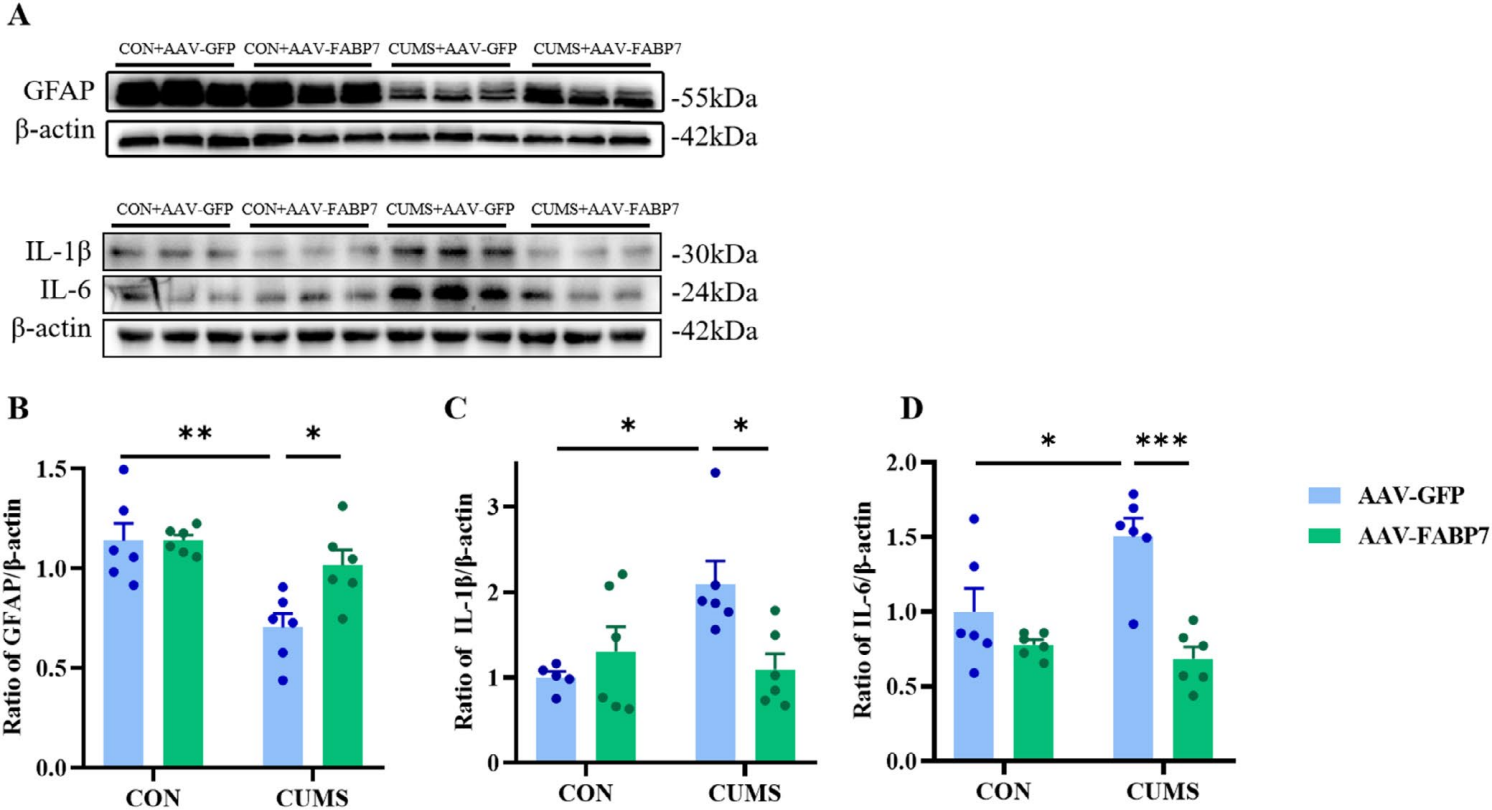
Effects of FABP7 overexpression on GFAP and inflammatory factor protein expression in HP of the CUMS mice. (A) Representative Western blots for GFAP, IL‐1β, and IL‐6. (B–D) Quantitative analysis of GFAP (B), IL‐1β (C), and IL‐6 (D) proteins in the HP. Data were presented as mean ± SEM (*n* = 5–6) and normalized to the control group (CON+AAV‐GFP) for analysis. Two‐way ANOVA with Tukey's honestly significant difference (HSD), *p < 0.05, **p < 0.01, ***p < 0.001.

To further extend the aforementioned results, changes in protein expression of Cav‐1 (*F*_CUMS(1,20)_ = 35.47, *p* < 0.001; *F*_FABP7(1,20)_ = 25.45, *p* < 0.001; *F*_CUMS×FABP7(1,20)_ = 6.280, *p* < 0.05; Figure 3H) and GFAP (*F*_CUMS(1,20)_ = 5.023, *p* < 0.05; *F*_FABP7(1,20)_ = 16.07, *p* < 0.001; *F*_CUMS×FABP7(1,20)_ = 4.922, *p* < 0.05; Figure 5A,B) in HP were examined by Western blotting. FABP7 overexpression in HP led to increased Cav‐1 expression (*p* < 0.001) and increased GFAP (*p* < 0.05) expression in HP in CUMS‐treated mice. To further verify the impact of FABP7 overexpression in HP on the regulation of depressive‐like behaviors after CUMS is related to actions in astrocytes, the effect of FABP7 overexpression on the co‐localization of FABP7 and GFAP was examined using immunofluorescence. We found that CUMS reduced the number of FABP7‐GFAP cells in the CA1 (*t*_(4)_ = 18.79, *p* < 0.001; Figure 4A,C), CA3 (*t*_(4)_ = 4.152, *p* < 0.05; Figure 4A,D), and DG (*t*_(4)_ = 2.850, *p* < 0.05; Figure 4A,E) subregions of the HP, and FABP7 overexpression reversed the phenomenon in the CA1 (*t*_(4)_ = 4.745, *p* < 0.01; Figure 4B,F), CA3 (*t*_(4)_ = 14.67, *p* < 0.001; Figure 4B,G), and DG (*t*_(4)_ = 3.650, *p* < 0.05; Figure 4B,H) subregions of the HP, which further validated the astrocyte specificity of FABP7 in the mechanism of depression.

### 3.8 FABP7 Overexpression in HP Ameliorates CUMS‐Induced Inflammatory Responses

These experiments show that CUMS can activate the expression of inflammatory factors. However, the effect of FABP7 overexpression in HP on the neuroinflammatory response in CUMS‐treated mice remains to be explored. Therefore, in this study, the protein expression levels of IL‐1β (*F*_CUMS(1,19)_ = 2.267, *p* = 0.1486; *F*_FABP7(1,19)_ = 3.621, *p* = 0.0723; *F*_CUMS×FABP7(1,19)_ = 7.843, *p* < 0.05; Figure 5A,C) and IL‐6 (*F*_CUMS(1,20)_ = 22.72, *p* < 0.001; *F*_FABP7(1,20)_ = 3.516 *p* = 0.0754; *F*_CUMS×FABP7(1,20)_ = 7.513, *p* < 0.05; Figure 5A,D) in HP were detected by Western blotting. Tukey's HSD confirmed that the levels of IL‐1β (*p* < 0.05) and IL‐6 (*p* < 0.001) in HP of the CUMS‐treated mice were reduced after overexpression of FABP7 in HP, indicating that FABP7 overexpression in HP improved the inflammatory response caused by CUMS.

### 3.9 FABP7 Overexpression Rescues CUMS‐Impaired Spinogenesis in the Hippocampus

**FIGURE 6:**
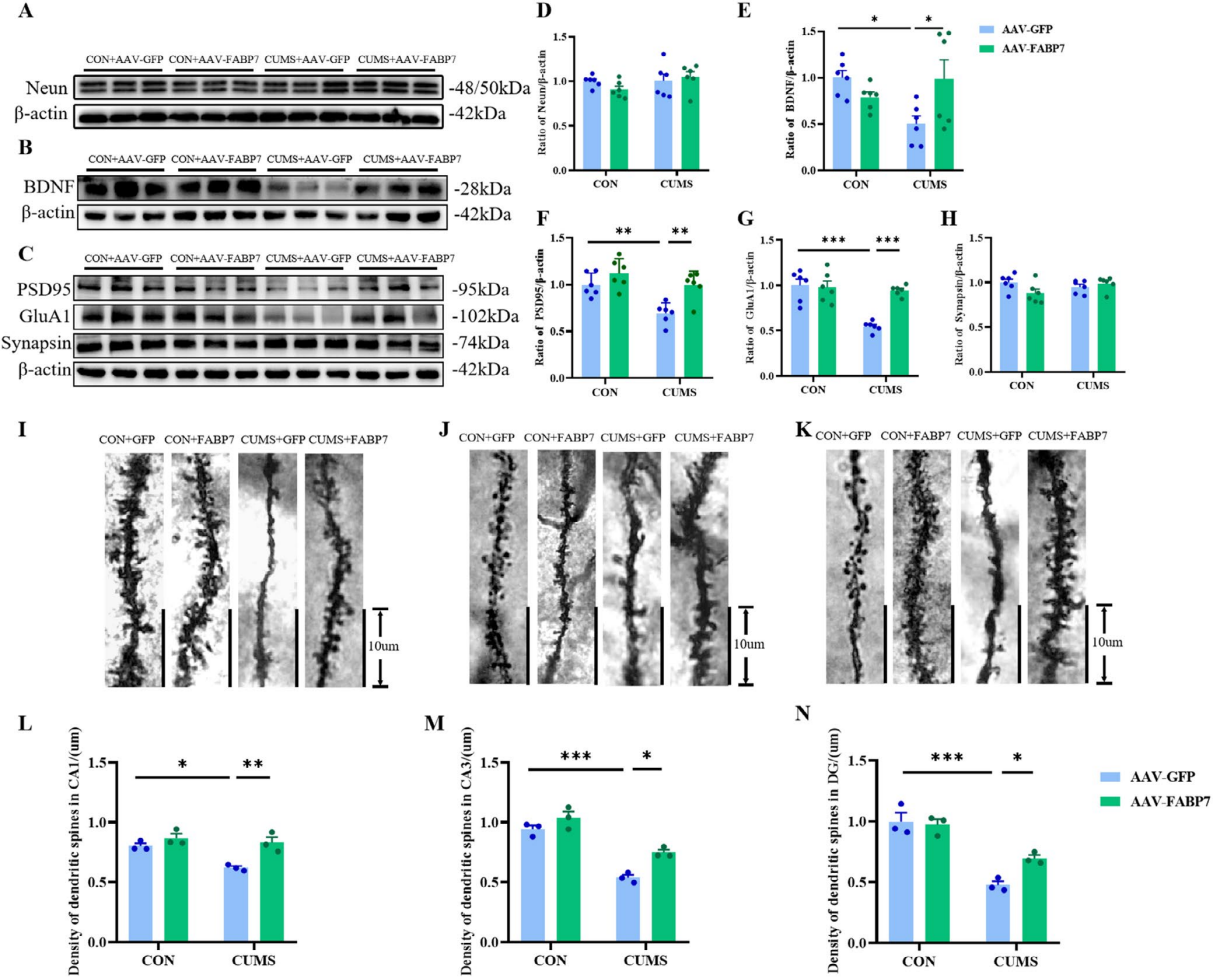
Effects of FABP7 overexpression on spinogenesis in HP of the CUMS mice. (A–C) Representative Western blots for NeuN (A), BDNF (B), PSD95, GluA1, and synapsin (C). (D–H) Quantitative analysis of NeuN (D), BDNF (E), PSD95 (F), GluA1 (G), and synapsin (H) proteins in the HP (*n* = 6 per group). (I–K) Representative images of dendritic spines from the HP CA1 (I), CA3 (J), and DG (K) subregions. Scale bars: 10 μm. (L–N) Quantitative analysis of dendritic spine density in the HP CA1 (L), CA3 (M), and DG (N) subregions (*n* = 3). Data were presented as mean ± SEM and normalized to the control group (CON+AAV‐GFP) for analysis. Two‐way ANOVA with Tukey's honestly significant difference (HSD), *p < 0.05, **p < 0.01, ***p < 0.001.

In the initial experiment presented here, CUMS caused a decrease in spinogenesis in HP. However, the influence of FABP7 overexpression in HP on spinogenesis in CUMS mice remains to be explored. Therefore, the protein expression levels of NeuN (*F*_CUMS(1,20)_ = 0.1959, *p* = 0.6628; *F*_FABP7(1,20)_ = 1.851, *p* = 0.1888; *F*_CUMS×FABP7(1,20)_ = 1.631, *p* = 0.2162; Figure 6A,D), BDNF (*F*_CUMS(1,20)_ = 1.279, *p* = 0.2714; *F*_FABP7(1,20)_ = 1.546, *p* = 0.2281; *F*_CUMS×FABP7(1,20)_ = 8.064, *p* < 0.05; Figure 6B,E), and the synapse‐related proteins PSD95 (*F*_CUMS(1,20)_ = 14.35, *p* < 0.01; *F*_FABP7(1,20)_ = 14.98, *p* < 0.01; *F*_CUMS×FABP7(1,20)_ = 2.62, *p* = 0.1212; Figure 6C,F), GluA1 (*F*_CUMS(1,20)_ = 13.48, *p* < 0.01; *F*_FABP7(1,20)_ = 22.42, *p* < 0.001; *F*_CUMS×FABP7(1,20)_ = 16.59, *p* < 0.001; Figure 6C,G), and synapsin (*F*_CUMS(1,20)_ = 1.208, *p* = 0.2848; *F*_FABP7(1,20)_ = 0.6569, *p* = 0.4272; *F*_CUMS×FABP7(1,20)_ = 4.518, *p* < 0.05; Figure 6C,H) in HP were examined by Western blotting. Tukey's HSD showed that after FABP7 overexpression in HP, the protein expression of BDNF (*p* < 0.05), PSD95 (*p* < 0.01), and GluA1 (*p* < 0.001) in HP of the CUMS‐treated mice showed a notable increase, while NeuN (*p* = 0.9339) and synapsin (*p* = 0.8856) remained unchanged.

To further analyze spinogenesis, the density of neuronal dendritic spines in the CA1 (*F*_CUMS(1,8)_ = 17.39, *p* < 0.01; *F*_FABP7(1,8)_ = 11.27, *p* < 0.05; *F*_CUMS×FABP7(1,8)_ = 4.816, *p* = 0.0595; Figure 6I,L), CA3 (*F*_CUMS(1,8)_ = 18.56, *p* < 0.01; *F*_FABP7(1,8)_ = 97.16, *p* < 0.001; *F*_CUMS×FABP7(1,8)_ = 2.641, *p* = 0.1428; Figure 6J,M), and DG (*F*_CUMS(1,8)_ = 4.017, *p* = 0.0800; *F*_FABP7(1,8)_ = 69.29, *p* < 0.001; *F*_CUMS×FABP7(1,8)_ = 6.582, *p* < 0.05; Figure 6K,N) subregions of the HP using Golgi‐Cox staining. Tukey's HSD confirmed that dendritic spine density in the CA1 (*p* < 0.05), CA3 (*p* < 0.001), and DG (*p* < 0.001) subregions of the HP was diminished in mice exposed to CUMS (Figure 6I–N). After FABP7 overexpression in the HP, the results were reversed in the CA1 (*p* < 0.01), CA3 (*p* < 0.05), and DG (*p* < 0.05) brain regions of CUMS‐treated mice. The above results indicate that FABP7 overexpression in HP can effectively improve the impairment in spinogenesis caused by CUMS.

### 3.10 FABP7 Overexpression in HP Modulates BBB‐Related Proteins in CUMS Mice

**FIGURE 7:**
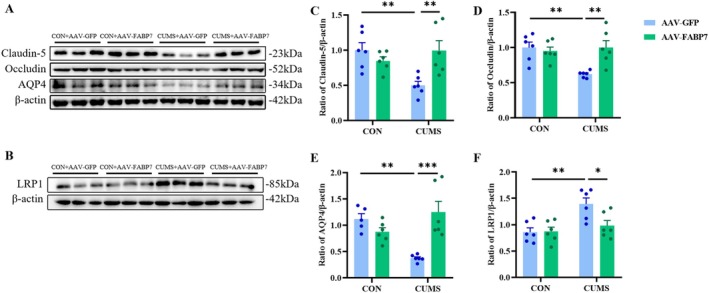
Effects of FABP7 overexpression on BBB stability‐related proteins in HP of the CUMS mice. (A, B) Representative Western blots for Claudun‐5, occludin, AQP4 (A), and LRP1 (B) in the HP. (C–F) Quantitative analysis of Claudun‐5 (C), occludin (D), AQP4 (E), and LRP1 (F) proteins in the HP. Data were presented as mean ± SEM (*n* = 5–6 per group) and normalized to the control group (CON+AAV‐GFP) for analysis. Two‐way ANOVA with Tukey's honestly significant difference (HSD), *p < 0.05, **p < 0.01, ***p < 0.001.

Astrocytes are involved in the composition of the BBB, which is strongly associated with the production of depression‐like behaviors. In a TBI model, FABP7 induces endothelial Cav‐1 overexpression to produce neurovascular protection [27]. Western blotting was conducted to investigate changes in protein expression of CLDN‐5 (*F*_CUMS(1,20)_ = 3.121, *p* = 0.0926; *F*_FABP7(1,20)_ = 3.290, *p* = 0.0847; *F*_CUMS×FABP7(1,20)_ = 10.90, *p* < 0.01; Figure 7A,C), occludin (*F*_CUMS(1,20)_ = 5.493, *p* < 0.05; *F*_FABP7(1,20)_ = 5.495, *p* < 0.05; *F*_CUMS×FABP7(1,20)_ = 9.352, *p* < 0.01; Figure 7A,D), AQP4 (*F*_CUMS(1,19)_ = 6.441, *p* < 0.05; *F*_FABP7(1,19)_ = 2.240, *p* = 0.1509; *F*_CUMS×FABP7(1,19)_ = 20.52, *p* < 0.001; Figure 7A,E), and LRP1 (*F*_CUMS(1,20)_ = 4.361, *p* < 0.05; *F*_FABP7(1,20)_ = 11.33, *p* < 0.01; *F*_CUMS×FABP7(1,20)_ = 4.930, *p* < 0.05; Figure 7B,F), indicators of BBB stability, in the HP. CUMS treatment decreased expression of tight junction (TJ) proteins CLDN‐5 (*p* < 0.01) and occludin (*p* < 0.01), as well as AQP4 (an aquaporin water channel protein) (*p* < 0.01), in the HP. In contrast, the expression of LRP1, a protein involved in water transport function, was increased (*p* < 0.01), suggesting that CUMS induces a reduction in BBB stability. After FABP7 overexpression in the HP, the expression of CLDN‐5 (*p* < 0.01), occludin (*p* < 0.01), and AQP4 (*p* < 0.001) was increased, while the expression of LRP1 was decreased in the HP of CUMS‐treated mice (*p* < 0.05). These findings suggest that overexpression of FABP7 in the HP ameliorates CUMS‐induced BBB damage.

## 4 Discussion

This study revealed the regulatory function of FABP7 expression in CUMS‐induced depressive‐like behavior. FABP7 overexpression in HP mitigated depressive‐like behaviors, ameliorated inflammatory responses triggered by CUMS, enhanced spinogenesis and function, and mitigated BBB damage.

The expression of FABP7 in astrocytes is significantly reduced in depression models induced by acute reserpine administration and chronic corticosterone administration [32]. In this study, a decrease in the protein expression of FABP7 in the CUMS depression model was observed. Past studies have confirmed that FABP7 is expressed in HP astrocytes of adult mice but not in mature neurons [12]. A notable diminution in the number of astrocytes and GFAP expression has been documented in HP in major depressive disorder (MDD) patients [33]. Similar evidence has also been reported in CUMS‐induced depressive rodent models [34, 35]. FABP7 regulates lipid raft function via modulating Cav‐1, a crucial component of lipid rafts involved in the response of astrocytes to external stimuli [18, 19]. Our results indicate that CUMS reduces the expression of GFAP and Cav‐1. This suggests that both FABP7 and Cav‐1 participated in the development of depressive‐like behaviors in CUMS mice.

Disruptions in BBB stability have been implicated in the onset of depressive disorders [36]. Additionally, inflammatory responses and alterations in spinogenesis represent classic mechanisms that drive the onset of depression. Whether FABP7 modulates depression‐like behavior caused by CUMS by modulating these classical processes needs further investigation. Therefore, adeno‐associated viruses were injected into the HP of mice to achieve overexpression of FABP7. FABP7 overexpression in HP reversed the reduction of Cav‐1 and GFAP in CUMS‐treated mice. In addition, behavioral experiments showed that FABP7 overexpression markedly decreased immobility in FST and TST and enhanced sucrose preference in CUMS‐treated mice. These results suggest that FABP7 overexpression in HP ameliorates the depression‐like actions of CUMS mice.

Stress‐induced activation of inflammation is a key factor in the development of depression pathophysiology. CUMS raises the levels of inflammatory factors in rat HP [37], and the increase in inflammatory cytokines induces depressive‐like behaviors [38]. In this research, CUMS increased IL‐1β and IL‐6 protein expression in HP, suggesting that the depressive‐like behavior in the CUMS mouse model is associated with the activation of neuroinflammation within HP. Research shows that astrocyte FABP7 may play an anti‐inflammatory role [8]. FABP7 overexpression in HP was shown here to reduce the levels of IL‐1β and IL‐6 in HP of CUMS mice, suggesting that FABP7 overexpression in HP inhibits the neuroinflammatory response after CUMS that contributes to the development of depressive‐like behavior.

CUMS is able to inhibit spinogenesis in HP, including reduced levels of neurotrophic factors and impaired spinogenesis [39, 40]. BDNF in the CNS is mainly synthesized in neuronal cells and can also be produced by astrocytes [41, 42]. In the present findings, CUMS did not affect the number of nuclei in mature neurons but resulted in a reduction of GFAP and a decrease in BDNF levels. FABP7 overexpression upregulated the expression of GFAP and BDNF in mice after CUMS, and it could be speculated that the reduction in BDNF may be caused by a decrease in the astrocyte number or impaired astrocyte function. Astrocytes can secrete BDNF, and FABP7 overexpression may directly increase BDNF expression by upregulating GFAP expression and increasing the number of astrocytes. On the other hand, astrocytes contribute to the maintenance of synaptic transmitter homeostasis, which may indirectly affect BDNF expression coming from neurons. In connection with the previous results, FABP7 overexpression may ameliorate the impaired function of astrocytes by inhibiting the inflammatory response in the CUMS model, promoting the maintenance of a dynamic balance of glutamate concentrations and an increase in the level of BDNF released from nerve terminal synapses. Considering that overexpression of FABP7 exerted no substantial influence on GFAP and BDNF levels in the control samples, it appears more likely that FABP7 affects the release of BDNF from synapses at neuronal terminals in an indirect manner.

Clinical and basic studies have shown that synaptic deficits in the HP cause depressive symptoms to arise [43]. Chronic stress can disrupt the synaptic structure of HP neurons and reduce dendritic branching [44]. The present results show that CUMS reduces the expression of PSD95 and GluA1 in HP but has no effect on the expression of synapsin. This suggests that the reduction in synapse‐related proteins may not be related to changes in the number of neurons but rather that neuronal cells are altered in their fine structure. Astrocytes regulate synapse formation, maturation, and signaling by secreting BDNF, which regulates synaptic plasticity [45, 46]. Impaired astrocyte function decreases the number of AMPA receptors, leading to impairment of synaptic plasticity and generation of depressive‐like behaviors in HP neurons [47, 48]. CUMS may impair the trophic support of neuronal synaptic structures by astrocytes through inhibition of astrocyte BDNF expression, resulting in alterations in neuronal microstructure and reduced expression of PSD95 and GluA1. A decrease in the number of excitatory synapses was observed in neurons co‐cultured with FABP7‐KO astrocytes and in the mPFC of FABP7‐KO mice, which was accompanied by a decrease in PSD95 protein expression [11]. Here, FABP7 overexpression improved the CUMS‐induced decline in PSD95 and GluA1 expression, which are proteins associated with excitatory synapses, suggesting that the ameliorative effect of FABP7 overexpression on depressive‐like behaviors in the CUMS model may be related to enhancement of excitatory synaptic transmission.

In vitro experiments revealed that neuronal cells co‐cultured with FABP7‐KO astrocytes had abnormal dendritic structure and reduced dendritic spine density [11], suggesting that FABP7 expression affects dendritic spine dynamics. Consistent with this finding, here FABP7 overexpression reversed the decline of dendritic spine density in CA1, CA3, and DG regions of mice induced by CUMS. This suggests that the ameliorative effect of FABP7 overexpression on depressive‐like behavior in the CUMS model results from the normalization of HP spinogenesis.

The disruption of BBB stability and integrity results in cerebral disturbances, subsequently facilitating the onset of psychiatric conditions, including depression [36, 49]. CUMS leads to decreased expression of tight junction proteins (TJs) claudin‐5 and occludin in the cerebrovascular system of mice, accompanied by increased BBB permeability and neuroinflammatory responses [50]. Alterations in the levels of functional proteins LRP1 and AQP4, as well as structural proteins ZO‐1 and occludin, lead to the activation of neuroinflammation, subsequently compromising the stability of the blood‐brain barrier [51]. Neuron‐targeted Cav‐1 partially treats traumatic brain injury by causing functional neuroplastic changes [52]. In a TBI model, FABP7 regulates BBB function in part by modulating Cav‐1 expression, affecting the intracerebral environment and BBB stability through astrocyte‐endothelial cell interactions [26]. The present results show that AQP4, CLDN‐5, and occludin expression are reduced, while LRP1 expression is increased in the HP of CUMS‐treated mice. The impact of CUMS on protein expression as described above is reversed after FABP7 overexpression. This suggests that the ameliorative effect of FABP7 overexpression in HP on CUMS‐induced depressive‐like behavior is connected with the protection of BBB stability and integrity.

Although a clear pattern of effects has been shown here for mechanisms implicated in depression and those involving FABP7, this study also has certain limitations. In vitro experiments revealed that exogenous DHA can activate PPAR in astrocytes to bind to its response elements, promoting FABP7 expression [13]. Detailed mechanisms of FABP7 in the intracellular trafficking of fatty acids and regulation of PPAR signaling to promote neuroplasticity remain to be further investigated.

In summary, this study suggests that FABP7 exerts an antidepressant effect in the CUMS‐induced depressive mice. This effect involves the modulation of Cav‐1 and GFAP expression in HP, the suppression of neuroinflammatory factors, an increase in spinogenesis, and the protection of BBB stability. These results provide a new theoretical foundation for the development of therapeutic targets for depression based on the modulation of FABP7 function.

## Author Contributions

**Bingjin Li:** conceptualization; supervision; funding acquisition; project administration; writing – original draft, review, and editing; **Zihui Geng:** investigation; data curation; formal analysis; software; visualization; writing – original draft. **Fanzhen Peng, Ziqian Cheng, Jingyun Su, Jinfang Song, Xu Han, and Runxin Li:** investigation; software; visualization; methodology; Ranji Cui and Xin Li: validation; writing – review and editing.

## Ethics Statement

Animal experiments were approved by the Committee of Jilin University.

## Conflicts of Interest

The authors declare no conflicts of interest.

## Supporting information


**Data S1** Supporting Information.

## Data Availability

All data generated or analyzed during this study are included in this published article.
